# The mapping of cortical activation by near-infrared spectroscopy might be a biomarker related to the severity of fibromyalgia symptoms

**DOI:** 10.1038/s41598-021-94456-2

**Published:** 2021-08-03

**Authors:** Daniela Gabiatti Donadel, Maxciel Zortea, Iraci L. S. Torres, Felipe Fregni, Wolnei Caumo

**Affiliations:** 1 Post-Graduation Program in Medical Sciences, School of Medicine, Universidade Federal do Rio Grande do Sul (UFRGS), Porto Alegre, Brazil; 2 Laboratory of Pain and Neuromodulation at Hospital de Clínicas de Porto Alegre (HCPA), UFRGS, Ramiro Barcelos, 2350, Bairro Rio Branco, Porto Alegre, RS CEP 90035-003 Brazil; 3 Pharmacology of Pain and Neuromodulation: Pre-Clinical Investigations Research Group at Hospital de Clínicas de Porto Alegre (HCPA), Porto Alegre, Brazil; 4 Department of Neurology, Berenson-Allen Center for Noninvasive Brain Stimulation, Beth Israel Deaconess Medical Center, Physical Medicine and Rehabilitation, Harvard Medical School, Boston, USA; 5 Pain and Palliative Care Service, Hospital de Clínicas de Porto Alegre (HCPA), Porto Alegre, Brazil; 6 Pain and Anesthesia in Surgery Department, School of Medicine, Universidade Federal do Rio Grande do Sul (UFRGS), Porto Alegre, Brazil

**Keywords:** Rheumatic diseases, Fibromyalgia, Neuroscience

## Abstract

The delta value of oxyhemoglobin (Δ-HbO) determined by functional near-infrared spectroscopy at prefrontal cortex (PFC) and motor cortex (MC) based on primary (25 °C) and secondary (5 °C) thermal stimuli presented a larger peak latency at left MC in fibromyalgia than in controls. The difference between HbO concentration 15 s after the thermal stimuli ending and HbO concentration before the thermal stimuli onset (Δ-HbO*) at left PFC increased 47.82% in fibromyalgia and 76.66% in controls. This value had satisfactory discriminatory properties to differentiate cortical activation in fibromyalgia versus controls. A receiver operator characteristics (ROC) analysis showed the Δ-HbO* cutoffs of − 0.175 at left PFC and − 0.205 at right PFC offer sensitivity and specificity of at least 80% in screening fibromyalgia from controls. In fibromyalgia, a ROC analysis showed that these cutoffs could discriminate those with higher disability due to pain and more severe central sensitization symptoms (CSS). The ROC with the best discriminatory profile was the CSS score with the Δ-HbO* at left PFC (area under the curve = 0.82, 95% confidence interval = 0.61–100). These results indicate that cortical activation based on Δ-HbO* at left PFC might be a sensitive marker to identify fibromyalgia subjects with more severe clinical symptoms.

## Introduction

Fibromyalgia is a chronic primary pain condition recently defined as nociplastic^1^ and described as diffuse pain associated with significant emotional distress related to functional disability that cannot be better accounted for by another chronic pain condition^1^. Other symptoms that accompany pain include sleep disturbance, joint stiffness, headache, abdominal discomfort, cognitive impairment, and depressive symptoms^2^. It is a prototypical condition of central sensitivity syndrome (CSS)^2,3^ which encompasses widespread pain and a persistent state of high reactivity that amplifies nociceptive stimuli^4^. The CSS cluster of symptoms includes psychological distress, sleep disturbances, fatigue, pain, allodynia, hyperalgesia, and expansion of the receptive field^5^. Although the mechanisms underpinning CSS are not fully elucidated, it is characterized by hyperexcitability by impaired functioning of neurons and circuits in nociceptive pathways, with an increase in neuronal excitability and synaptic efficacy and reduced inhibition.

Human models of central sensitization studies confirm that this facilitatory system becomes active and underpins the maintenance of the centrally sensitized state^6,7^. As the experience of chronic pain is associated with activity in multiple networks in the central nervous system (CNS), chronic pain is considered a CNS disorder^8^. As a result of multi-network activation in the CNS, chronic pain comprises multiple components, including sensory, emotional, cognitive, and behavioral elements^9^. To date, the focus has been to measure functional correlates of the human pain experience using blood flow-based methods, such as positron emission tomography (PET); electrophysiological methods, such as magnetoencephalography (MEG) and electroencephalography (EEG); or functional magnetic resonance imaging (fMRI) and functional near-infrared spectroscopy (fNIRS). fMRI and fNIRS measure brain activity by detecting changes associated with transient dynamics of the vascular response by blood oxygenation level-dependent (BOLD). These neuroimage exams can record the signal from all brain regions, unlike EEG/MEG, which are biased toward the cortical surface^10^. While fMRI has a higher spatial resolution, but poorer temporal resolution compared with EEG, fNIRS assesses the coupling between cerebral blood flow and neuronal activation with better temporal resolution than fMRI. fNIRS is a relatively flexible technology that provides detailed biochemical specificity in changes in both oxyhemoglobin (HbO) and deoxyhemoglobin to give insight into the BOLD response's physiological mechanisms. It is portable and may be beneficial in experimental paradigms that are not well suited to the fMRI scanner, such as face-to-face communication^11^.

Up to now, several meta-analyses summarize findings applied to the field of pain, and several studies have investigated the cerebral changes associated with chronic pain^12^. They focus on structural^13,14^, functional^15,16^ and neurochemical brain alterations^17,18^. These studies provide a powerful strategy to identify convergent brain regions altered in pain processing in chronic pain. According to a previous study, augmented cerebral activation upon painful stimulation might contribute to fibromyalgia pain^19^. Another study with fibromyalgia found lower motor cortex (MC) activation during repetitive movement of the finger-tapping task at pressing a push-button panel with the right-hand thumb in two modalities—slow and fast^20^. Again, in an earlier study in healthy subjects, morphine attenuated the pain signal measured by fNIRS in the medial Brodmann’s area 10 and primary somatosensory cortex (S1)^21^. In comparison, fibromyalgia patients showed an increase in HbO at the dorsolateral prefrontal cortex (PFC) upon painful stimulation compared to healthy controls^19^. Such studies found correlations between pain stimulus intensity and magnitude of activation of cortical areas involved in pain processing^16,22^. These areas include contralateral SI and bilateral second (SII) somatosensory cortices^23^ and the PFC^24^.

PFC and MC are regions that have received attention mainly as targets for non-invasive brain stimulation using electrical stimuli applied over the scalp [e.g., transcranial magnetic stimulation (TMS) and transcranial direct current stimulation (tDCS)] to treat pain and psychiatric disorders. However, there is a gap in the literature regarding comprehending how cortical activation at these specific areas relates to emotion [i.e., PFC], motor function (M1)^25,26^ clinical symptoms of central sensitization and disability due to pain. Thus, in the current study, we compared the cortical activation patterns in fibromyalgia subjects and controls-based brain cortical activation after immersing the right hand into water at two different temperatures 25 °C or primary stimulus and 5 °C or secondary stimulus. We assessed the cortical activation by the peak latencies of oxyhemoglobin (HbO) (assessed in seconds) until maximum cortical amplitude of HbO at PFC and MC. We also assessed the cortical deactivation relative to decrease in the blood-oxygen-level-dependent (BOLD) fNRIS signal) assessed by difference from baseline HbO and HbO measurement 15 s after thermal stimuli ending, defined as Δ-HbO*. We also explored properties of Δ-HbO* at PFC to discriminate fibromyalgia subjects from controls and identify fibromyalgia subjects with more severe clinical symptoms of CSS and disability due to pain. We hypothesized that measuring cortical activation through fNIRS and comparing Δ-HbO and Δ-HbO* differences could be a sensitive marker to discriminate fibromyalgia subjects from controls and those with more severe symptoms related to fibromyalgia.

## Patients and methods

### Design overview, settings, and participants

The protocol of this cross-sectional study was approved by the Committee Board (Institutional Review Board at Hospital de Clínicas de Porto Alegre Ethics number 20170049), according to the Declaration of Helsinki. All participants provided written informed consent.

### Recruitment, inclusion, and exclusion criteria

We included right-handed literate women aged between 18 and 65 years recruited from the Chronic Pain Clinic and Basic Health Unit of the Hospital de Clínicas de Porto Alegre and referrals from other clinic units. Also, advertisements were posted in public places in Porto Alegre and on the internet. Thereafter, volunteers were contacted by phone and screened for eligibility. For the fibromyalgia group, participants had to have a confirmed diagnosis of fibromyalgia according to the American College of Rheumatology^2^. A skilled physician with experience in a pain clinic re-examined patient and confirmed the diagnosis. We excluded pregnant patients and women with a history of malignancy or uncompensated chronic diseases. The control group excluded participants who reported pain or frequent use of painkillers; pregnancy; clinical disease (e.g., diabetes, hypertension); a history of neuropsychiatric comorbidities; or use of benzodiazepines, anticonvulsants, or antidepressant drugs. Both groups were instructed to refrain from consuming stimulating drinks and alcohol at least 6 h before the assessments.

### Outcomes

The outcomes related to cortical activation were the peak latency of the HbO, assessed in seconds, considering the onset period of each of two thermal stimuli (primary at 25 °C and secondary at 5 °C) until reaching the cortical amplitude maximum at PFC and MC. Also, we assessed the cortical activation by difference in HbO concentration (mM) from baseline until the maximum cortical amplitude of each of two thermal stimuli (Δ-HbO). The cortical deactivation relative to decrease in the BOLD fNIRS signal assessed by difference from baseline HbO and HbO measurement 15 s after thermal stimuli ending, defined as Δ-HbO*. The disability due to pain and central sensitization symptoms were the primary clinical outcomes in the fibromyalgia group. For the analysis that related to the clinical outcomes, the study's primary interest factor was PFC activation assessed by Δ-HbO*.

### Instruments and assessments

#### Data acquisition

Cortical activation was evaluated by fNIRS, with a NirX NirScout 16 × 24 near-infrared spectroscopy device [scan rate of 3.91 Hz, dual-wavelength light-emitting diode sources (760 and 850 nm)] with 16 sources and 16 detectors spaced at 3 cm and placed over the scalp using the caps provided by EASYCAP^®^. The montage creates 40 channels and covers bilateral prefrontal and cortical motor areas. Probe localization was established using the international 10–10 EEG system. Imaging data were preprocessed using the Brain AnalyzIR^®^ software on the MATLAB (MathWorks, Natick, MA, USA) platform. Appendix I (Supplementary material) presents the relationship between fNIRS channels 1–20 and the EEG 10–10 system to compose the channels used.

#### Screening and preprocessing

Measurements for each subject were analyzed, and trials, channels, or participant data were rejected from further analysis in a two-step preprocessing protocol: first, by looking at time measures, and second, by the quality of the signals as assessed by artifact-detection algorithms (which either excluded the data of whole channels per infant or data from individual trials within a channel, according to the magnitude of the artifact). The criteria for channel rejection included measuring the coefficient of variation (CV) of the signal. Channels were excluded if the CV of the attenuation measurement for each wavelength exceeded 7.5%^27^.

**Figure 1:**
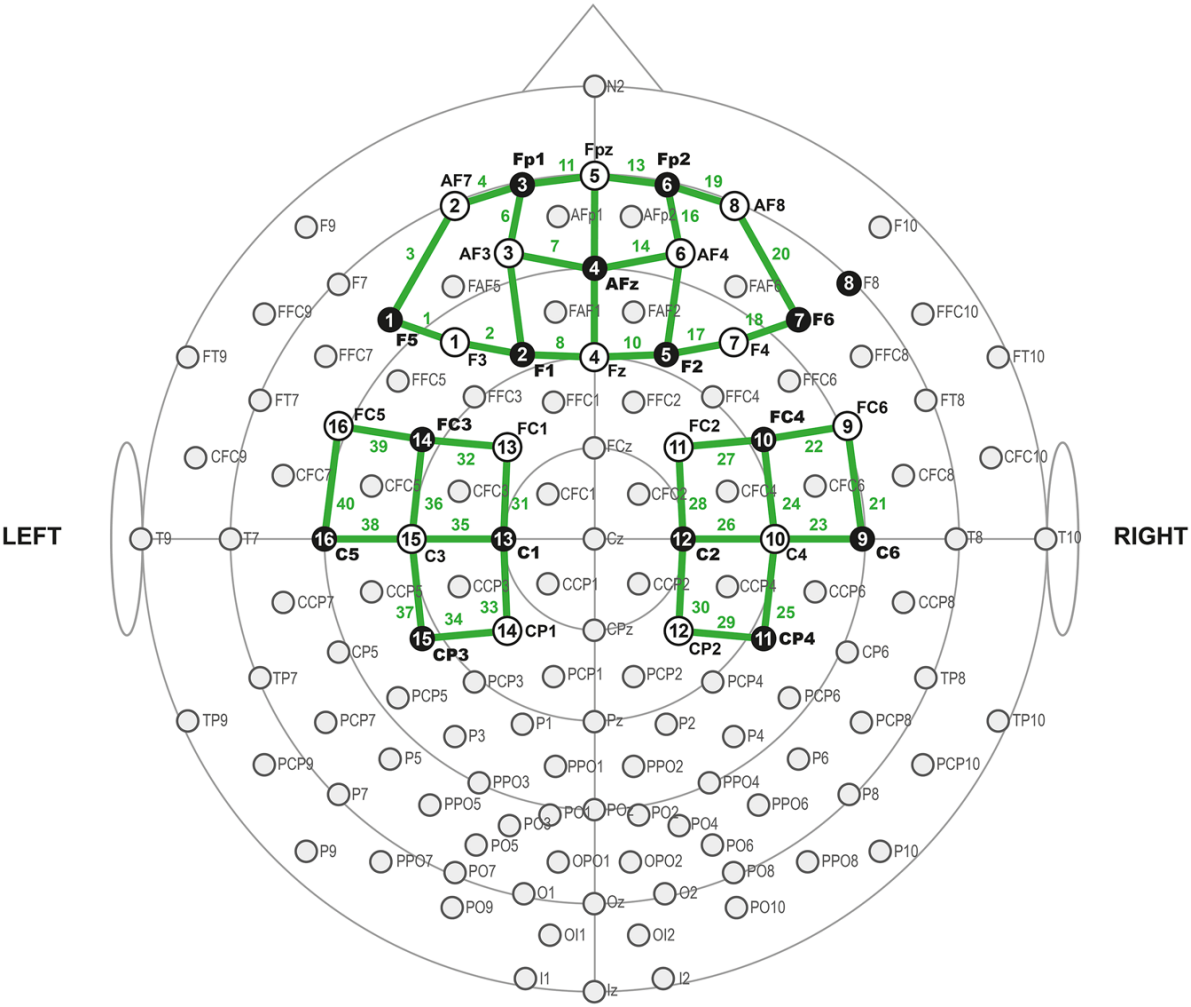
The fNIRS' optodes montage. Black dots represent detectors, and white dots represent sources. Left prefrontal cortex—channel 1–9 plus 11 and 12. Right prefrontal cortex—channel 12–20 plus Channel 9 and 10. Right motor cortex—channel 21–30. Left motor cortex—Chanel 31–40.

Data were band-passed from 0.01 to 0.2 Hz. Considering an inter-optode distance of 3 cm and a differential path length (DFP) of 7.25 and 6.38, the data were finally converted to HbO using the modified Beer-Lambert law and exported to MatLab. Based on previous studies^28^ we calculated the mean HbO baseline concentration as the it’s media during the 30 s before the primary stimulus and so, defined three main measures: (a) the latency (time in seconds) to reach the highest HbO concentrations after stimulus onset (peak latency); (b) The Δ-HbO was the difference in the HbO concentration (mM) from baseline until the cortical amplitude maximum of each of two thermal stimuli; (c) the difference of HbO concentrations between baseline and the 15 s after thermal stimuli ending (Δ-HbO*)^29^. We grouped channels in four cortical areas based on EEG 10/10 system parameters and Brodmann’s area (Fig. 1): left and right PFC and left and right MC.

#### Experimental paradigm

**Figure 2:**
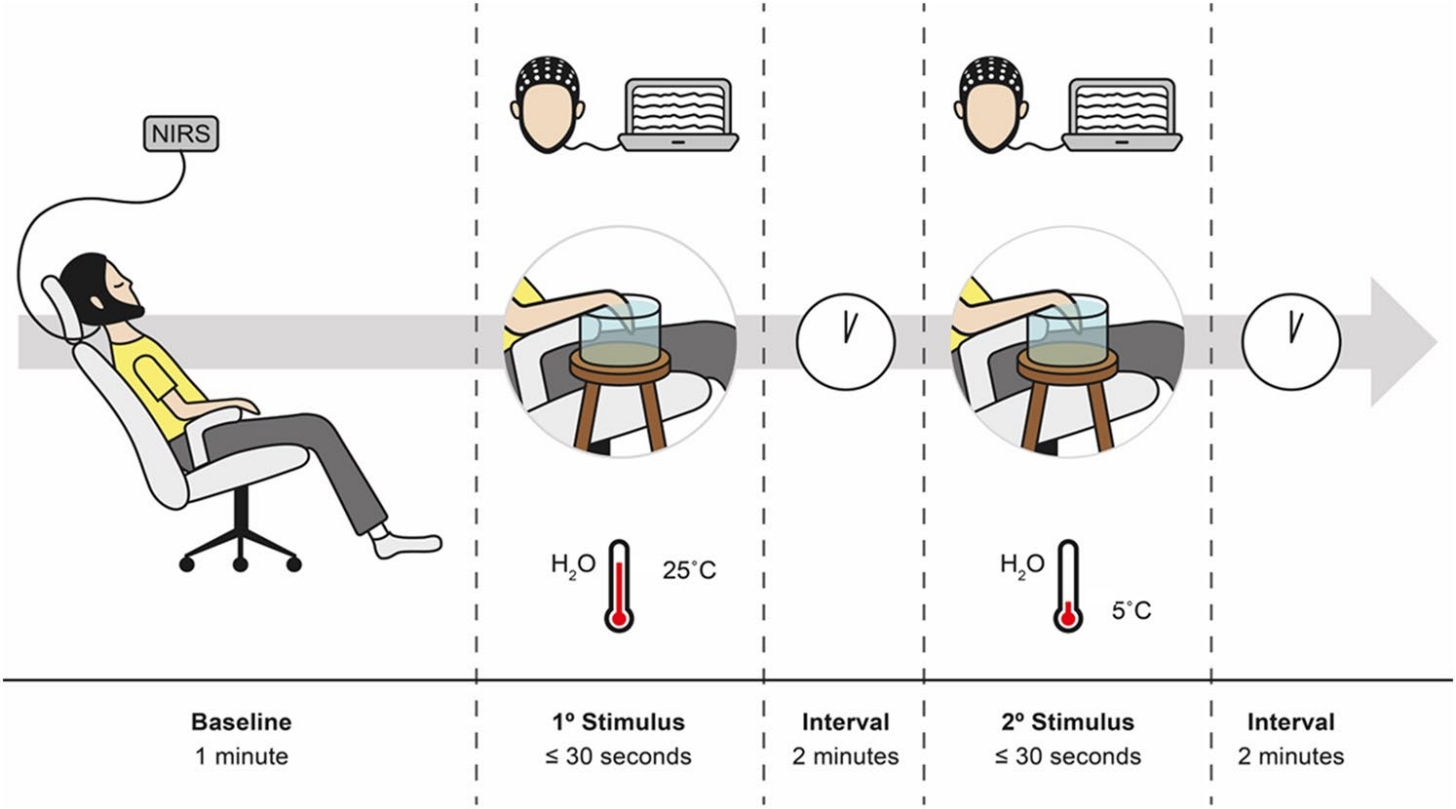
Timeline of the experimental paradigm. Timeline of each assessment pre- and post-thermal stimuli with primary stimulus (at 25 °C) and secondary stimulus (at 5 °C). The cold test was induced by the immersion of the right hand in the water for 30 s or until feeling the first pain sensation with both stimuli and a break of 2 min between each trial.

Subjects sat in a comfortable chair with their arm rested, lights turned off, and room temperature kept constant (around 21 °C). After explaining, demonstrating, and addressing concerns about each thermal stimulation, we asked subjects to keep their eyes closed and to reduce any kind of motor activity not related to the experiment. After verifying the correct positioning and adequate signal capture, we proceeded to data registering. Preliminarily we recorded 60 s in a resting state, during which the participant was requested to stay relaxed with open eyes, fixing on a point on the computer monitor. Thermal stimulation was based on cold pressor test and its capacity of reproducing tonic pain. It was performed while recording the cortical activation for posterior offline analysis. For the thermal test, the participants immersed their right hand in a bucket with water at two different temperatures: 25 °C (innocuous—primary stimulus) and 5 °C (noxious—secondary stimulus). They maintained their hand immersed for 30 s or until feeling the first pain sensation. After each thermal test (primary or secondary), the participant rested for 2 min. A digital thermometer measured the water temperature during all experiment time. Thus, we reduce the possible confound effect of different temperatures and other environmental stimuli that could produce cortical activation and influence in our experiment. The total time of the experimental paradigm was around 15–20 min. Figure 2 illustrates the paradigm used.

#### Assessment of pain disability due to pain and central sensitization symptoms

- The Brazilian Portuguese version of the Profile of Chronic Pain: Screen (BP-PCP:S)^29^ was used to assess the disability due to pain (DRP) for daily activities. It consists of 15 items (total score 0–91) in three parts: severity scale [two items in 6-point Likert scale and two in numeric scale (0–9 in intervals of 0.5 units), possible range 0–30], interference scale (six items in 6-point Likert scale; possible range 0–36), and emotional burden scale (five items in 5-point Likert scale; possible range 0–25).
- The Central Sensitization Inventory for Brazilian Population (CSI-BP) consists of 25 items (total score 0–100) assessing physical symptoms, emotional distress, headache/jaw symptoms, and urological symptoms. This scale allows a rapid tracking of symptoms associated with central sensitization to guide therapeutic strategies and indicate prognostic factors. Higher scores indicate a higher degree of self-reported symptomatology. Additionally, part B of SCI assesses the presence of psychiatric diagnosis and neurological disorders associated with central sensitization^30^.

#### Assessment of sociodemographic characteristics and other clinical and psychological measures

*The American College of Rheumatology*^2^ criteria was used in physician-administered and patient self-administered questionnaires, increasing the correct diagnoses. The scale of FM symptoms ranging from 0 to 3 and adding the widespread pain index (WPI) to the modified severity scale (SS scale). The WP quantifies the extent of bodily pain on a 0–19 scale by asking patients if they have had pain or tenderness in 19 different body regions (shoulder girdle, hip, jaw, upper arm, upper leg, lower arm, and lower leg on each side of the body. As well as the upper back, lower back, chest, neck, and abdomen) over the past week, with each painful or tender region scoring one point. The CSS scale quantifies symptom severity on a 0–12 scale by scoring problems with fatigue, cognitive dysfunction, and unrefreshed sleep over the past week, each on a scale from 0 to 3. Also, it assesses if there are problems at the last 6 months often present, such as life-disturbing problems, pain or cramps in the lower abdomen, depression symptoms, headache, etc.

We used the Beck Depression Inventory (BDI-II)^31^ for depressive symptoms, the State-Trait Anxiety Inventory—short version (STAI-SV)^3^, and the Brazilian Pain Catastrophizing Scale (BP-PCS)^32^ for sample characterization purposes. A standardized query was used to assess demographic data and medical comorbidities. Patients were requested to provide information about their age, sex, level of education, marital status, and lifestyle habits. They also provided information about their health status, including clinical and psychiatric diagnoses. Analgesic use was defined as an average of the number of days per week that analgesics were used during the previous month. For data analysis, analgesic use was included as a dichotomous variable (4 or fewer days per week, or > 4 days per week). This approach was chosen because analgesic use among patients with chronic pain changes each week, depending on their level of pain.

### Data analysis

All continuous variables were tested for normality using the Shapiro–Wilk test and box-plot diagrams. The majority presented an indication of non-parametric distributions. Therefore, for sample characterization, we used Mann–Whitney U tests for continuous variables and Fisher’s exact tests for categorical variables. We compared the effect of thermal stimuli through the speed on reaching the HbO peak (seconds) and by the difference of Δ-HbO pre- to post-stimuli within in each group (fibromyalgia or controls) and between groups^33^. Generalized Estimating Equations (GEE) models with an exchangeable working correlation were used to compare the group effect (fibromyalgia and controls) speed of activation (peak latency) and to compare the group effect on cortical deactivation based on Δ-HbO* value. In the models, interactions among the factors (group and temperature) were also examined. Effect sizes were reported according to Cramer’s V [for one degree of freedom (df), the effect size is classified as small (0.1), medium (0.3), or large (0.5)]^34^.

Spearman's rho coefficient was used to assess the relationship between left and right PFC activation assessed by the Δ-HbO* with the disability due to pain and central sensitization symptoms in fibromyalgia. After confirming the corresponding assumptions, a multivariate linear regression model was performed to adjust for multiple to assess the relationship between dependent variables [scores related to disability due to pain for daily activities (BP-PCP:S) and central sensitization scores (CSI-BP)] and the cortical activation level assessed by Δ-HbO* as the independent variable. All analyses were adjusted for multiple comparisons using Bonferroni's multiple comparison test. The area under the curves (AUCs) with exact binomial 95% confidence intervals (CI) are presented. The cutoff values with the highest Youden index, with 80% sensitivity and 80% specificity, are presented in each of four indexes, and all showed a receiver operator characteristics (ROC) AUC higher than 0.68.

For the analysis of an association between the cortical activation assessed by Δ-HbO* on PFC and fibromyalgia symptoms, a priori sample size estimation indicated a study of 20 subjects for type I and II error rates of 0.05 and 0.20, respectively, and anticipating an effect size of 0.6 for multiple regression analysis, which allows for two predictors (cortical activation level assessed by the Δ-HbO* on the left and the right PFC). Finally, considering the likely attrition rate and other unexpected factors, we increased the sample by 10%, and the required sample size was 22 patients^35^. All analyses considered a significance level of α < 0.05 for two-tailed tests. To analyze the data, we used the software SPSS version 22.0 (SPSS, Chicago, IL, USA).

## Results

### Sample characterization

A total of 46 volunteers were enrolled in the study, and 5 were excluded (1 from the fibromyalgia group due to problems with the fNIRS registration and 4 from the control group due to acute illness and use of antidepressants). The comparisons between groups related to sociodemographic and clinical characteristics are presented in Table 1. Groups differed significantly in all characteristics, in the sense that fibromyalgia subjects were older, with higher body mass index and fewer years of study. As expected, the fibromyalgia group showed more severe clinical symptoms and higher medication use. Data are presented in Table 1.

**Table 1:** Demographic and clinical characteristics for the total sample (n = 41).

	Fibromyalgia (n = 22)		Controls (n = 19)		*P* value
	Mean	SD	Mean	SD	
**Demographic variables**					
Age (years)	47.14	9.49	34.68	12.45	0.001
Body mass index^$^	27.83	4.17	23.12	3.37	0.001
Years of study	13.55	2.74	17.29	3.31	< 0.001
**Outcome’s variables**					
Portuguese Profile of Chronic Pain Screen (BP-PCP:S) total score	71.95	15.56	14.11	15.54	< 0.001
BP-PCP:S—severity	25.68	3.24	6.74	6.37	< 0.001
BP-PCP:S—activity	27.50	9.74	4.47	7.43	< 0.001
BP-PCP:S—emotional burden	18.77	5.00	2.89	4.01	< 0.001
Brazilian Portuguese Central Sensitization Inventory (BP-SCI)	71.18	13.72	21.37	11.19	< 0.001
**Clinical variables and psychological measures**					
The American College of Rheumatology (ACR)					
Widespread pain index (WPI)	13.81	22.50			
Symptom Severity (SS) Scale	10.58	1.58			
Beck Depression Inventory (BDI-II) total score	28.45	12.07	7.53	9.39	< 0.001
Strait-Trait Anxiety Inventory—short version (STAI-SV)—State scale	30.00	7.34	22.32	6.48	0.001
Strait-Trait Anxiety Inventory—short version (STAI-SV)—Trait scale^$^	27.81	3.44	18.11	4.53	< 0.001
Brazilian Pain Catastrophizing Scale (B-PCS) total score	39.27	8.40	7.37	12.57	< 0.001

Medication use	Yes/no	% Yes	Yes/no	% Yes	
**Psychiatric medication use**					
Benzodiazepine	4/18	18.2	–	–	–
Antidepressant	12/10	54.5	–	–	–
Anticonvulsant	10/12	45.5	–	–	–
Number of days of analgesic use per week in the previous month (< 4 times/> 4 times)	20/2	90.9	–	–	–

*SD* standard deviation.

All *P* values are based on Mann–Whitney test, except for “regular analgesic use” which is based on Fisher’s exact test. $ = missing data for FM group with n = 21.

**Figure 3:**
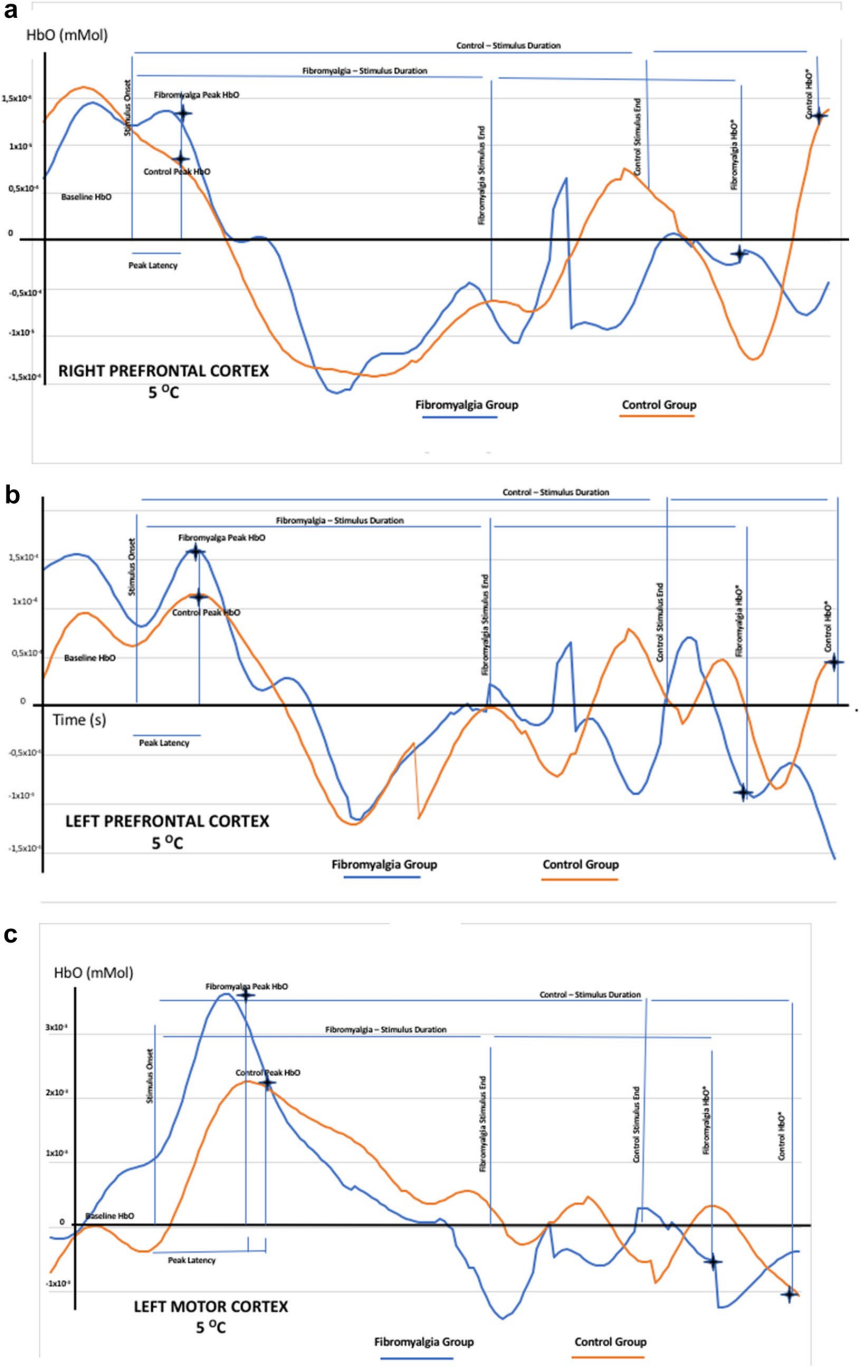
Representative waveforms from cortical activation at right PFC (**A**); left PFC (**B**) and left motor cortex (**C**). Figures shows waveforms from cortical activation of an illustrative subject with fibromyalgia or one control. They showed waveforms of oxyhemoglobin (HbO) by fNIRS upon to primary (25 °C) or secondary (5 °C) stimulus. The figure presents both sides left and right prefrontal cortex (PFC). (**A**) Peak latency represents the maximum peak (time in seconds) and HbO millimolar. (**B**) (ΔHbO) [steady-state HbO before thermal primary or secondary stimuli onset minus the absolute value of peak HbO] (mM). (**C**) The cortical activation 15 s after thermal stimuli end by the variability of absolute HbO concentration [the difference between the delta value of HbO (Δ-HbO) 15 s from thermal stimuli end and Δ-HbO before thermal stimuli onset (Δ-HbO*)].

Figure 3 shows representative waveforms of cortical response of both sides of the left and right prefrontal cortex (PFC). They show peak latency and data of HbO (Δ-HbO and Δ-HbO*) from one subject with fibromyalgia and one control subject submitted to the primary thermal stimulus (25 °C) or secondary thermal stimulus (5 °C).

### Cortical speed of activation assessed by HbO peak latency and compared on groups considering PFC and MC by primary and secondary thermal stimuli applied on right hand

The GEE models revealed a significant temperature effect in Δ-HbO* at both PFC and MC. The main effect indicated there is an increment on Δ-HbO* at 5 °C comparing to 25 °C on both PFC and MC. For the fibromyalgia group, at the left PFC the difference in absolute -Δ-HbO concentration at primary thermal stimulus (25 °C) versus secondary thermal stimulus (5 °C) was an increase equal to 47.82% [mean (SD) 0.23 (0.03)/0.34 (0.05)] compared to 76.66% in controls [mean (SD) 0.30 (0.05)/0.53(0.14)] (Wald χ^2^ = 5.33, df = 1, *P* = 0.02, Cramer’s V = 0.36. For the fibromyalgia group, at the right PFC the difference in absolute -Δ-HbO concentration at primary thermal stimulus (25 °C) versus secondary thermal stimulus (5 °C) was an increase equal to 54.54% [mean (SD) 0.22 (0.04)/0.34 (0.07)] compared to 52% in controls [mean (SD) 0.25 (0.05)/0.38 (0.08)] (Wald χ^2^ = 2.07, df = 1, *P* = 0.2, Cramer’s V = 0.35).

The GEE models revealed significant main effects for the group in the maximum cortical activation (peak latency) of HbO curve at left MC (Wald χ^2^ = 5.39, df = 1, *P* = 0.02, Cramer’s V = 0.36) (Table 2). However, we found no difference in either temperature effects or interaction between temperature and group. For the fibromyalgia group, the peak latency difference at primary thermal stimulus (25 °C) versus secondary thermal stimulus (5 °C) was an increase equal to 15.50% [mean (standard deviation [SD]) 5.61 (0.52)/6.48 (0.62)] compared to 1.11% in controls [mean (SD) 8.09 (0.90)/8.00 (0.73)], respectively. Therefore, cortical activation occurred at a slower speed in fibromyalgia patients than in controls.

**Table 2:** Generalized Estimating Equations (GEEs) to compare the cortical activation parameters induced by thermal stimulus (25 °C and 5 °C) on the bilateral PFC and MC between fibromyalgia patients and controls (n = 41).

	Fibromyalgia (n = 22)		Controls (n = 19)		Group effect		Temperature effect		Group * temperature interaction	
	25 °C	5 °C	25 °C	5 °C						
	Mean (SE)	Mean (SE)	Mean (SE)	Mean (SE)	Wald χ^2^	*P*	Wald χ^2^	*P*	Wald χ^2^	*P*
**Oxyhemoglobin (HbO) peak latency**										
PFC left	4.95 (0.51)	6.33 (0.71)	7.26 (0.79)	6.71 (0.81)	2.84	0.092	0.45	0.503	2.4	0.122
PFC right	5.73 (0.67)	5.74 (0.66)	7.08 (0.73)	6.07 (0.71)	1.08	0.298	0.82	0.365	0.84	0.359
MC left	5.61 (0.52)	6.48 (0.62)	8.09 (0.90)	8.00 (0.73)	5.39	**0.020**	0.63	0.427	0.95	0.331
MC right	5.36 (0.56)	5.40 (0.78)	6.98 (0.79)	5.53 (0.83)	1.00	0.317	1.41	0.235	1.56	0.212
**Delta value of oxyhemoglobin (mM) (ΔHbO)—from baseline to peak amplitude of the curve**										
PFC left	0.35 (0.05)	0.41 (0.05)	0.39 (0.10)	0.49 (0.10)	0.42	0.519	2.6	0.107	0.14	0.712
PFC right	0.35 (0.07)	0.44 (0.06)	0.32 (0.05)	0.44 (0.06)	0.05	0.818	3.17	0.075	0.08	0.777
MC left	0.39 (0.06)	0.57 (0.08)	0.44 (0.06)	0.72 (0.13)	1.24	0.265	6.90	**0.009**	0.27	0.603
MC right	0.35 (0.05)	0.45 (0.06)	0.50 (0.07)	0.44 (0.05)	1.48	0.223	0.12	0.729	2.13	0.144
Delta value of HbO concentration (mM) (ΔHbO*) [based on HbO concentration before thermal stimuli onset minus HbO concentration level 15 s after thermal stimuli end] at 5 °C and 25 °C										
PFC left	0.23 (0.03)	0.34 (0.06)	0.30 (0.05)	0.53 (0.14)	2.01	0.157	5.33	**0.021**	0.55	0.457
PFC right	0.22 (0.04)	0.34 (0.07)	0.25 (0.05)	0.38 (0.08)	0.3	0.581	5.07	**0.024**	0.02	0.889
MC left	0.17 (0.04)	0.37 (0.06)	0.28 (0.05)	0.54 (0.11)	3.05	0.081	13.15	**< 0.001**	0.22	0.637
MC right	0.15 (0.03)	0.24 (0.05)	0.26 (0.05)	0.35 (0.07)	3.7	0.054	3.82	0.051	< 0.01	0.952

Bold denotes data that actually had statistical difference and also the identification of each parameter - just in order to clarify data.

Data are presented as mean and standard error (SE) (n = 41).

*SE* standard error, *PFC* pre-frontal cortex area, *MC* motor cortex area, *HbO* oxyhemoglobin, *mM* millimolar.

The GEE models revealed no significant main effects for the group in Δ-HbO concentration (Wald χ^2^ = 0.45, df = 1, *P* = 0.50) or the interaction between temperature and group. However, we found a difference in the temperature effect in Δ-HbO at left MC (Wald χ^2^ = 6.90, df = 1, *P* = 0.009). For the fibromyalgia group, the Δ-HbO difference at primary thermal stimulus (25 °C) versus secondary thermal stimulus (5 °C) was an increase equal to 46.15% [mean (SD) 0.39 (0.06)/0.57 (0.08)] compared to 63.64% in controls [mean (SD) 0.44 (0.06)/0.72 (0.13)], respectively. Therefore, cortical activation due to thermal stimuli at left MC based on Δ-HbO was higher in controls than in fibromyalgia subjects.

The GEE models revealed a significant temperature effect in Δ-HbO* at both PFC and MC. The main effect indicated there is an increment on Δ-HbO* at 5 °C comparing to 25 °C on both PFC and MC. For the fibromyalgia group at the left PFC the Δ-HbO* difference at primary thermal stimulus (25 °C) versus secondary thermal stimulus (5 °C) was an increase equal to 47.82% [mean (SD) 0.23 (0.03)/0.34 (0.05)] compared to 76.66% in controls [mean (SD) 0.30 (0.05)/0.53(0.14)] (Wald χ^2^ = 5.33, df = 1, *P* = 0.02, Cramer’s V = 0.36). For the fibromyalgia group at the right PFC the Δ-HbO* difference at primary thermal stimulus (25 °C) versus secondary thermal stimulus (5 °C) was an increase equal to 54.54% [mean (SD) 0.22 (0.04)/0.34 (0.07)] compared to 52% in controls [mean (SD) 0.25 (0.05)/0.38 (0.08)] (Wald χ^2^ = 2.07, df = 1, *P* = 0.2, Cramer’s V = 0.35). Therefore, cortical late response at left PFC based on Δ-HbO* was higher in controls than in fibromyalgia.

For the fibromyalgia group, at the left MC PFC the Δ-HbO* difference at primary thermal stimulus (25 °C) versus secondary thermal stimulus (5 °C) was an increase equal to 117.64% [mean (SD) 0.17 (0.04)/0.37 (0.06)] compared to 92.85% in controls [mean (SD) 0.28 (0.05)/0.54 (0.11)] (Wald χ^2^ = 13.15, df = 1, *P* = 0.02, Cramer’s V = 0. 57). For the fibromyalgia group at the right MC PFC the Δ-HbO* difference at primary thermal stimulus (25 °C) versus secondary thermal stimulus (5 °C) was an increase equal to 60% [mean (SD) 0.15 (0.03)/0.24 (0.05) compared to 34.62% in controls [mean (SD) 0.26 (0.05)/0.35 (0.07)] (Wald χ^2^ = 3.82, df = 1, *P* = 0.05, Cramer’s V = 0.30). Therefore, fibromyalgia subjects showed lower deactivation at the left MC by Δ-HbO* 15 s after thermal stimulation end compared to controls. We did not find an interaction effect between groups and temperature on the deactivation response at both the left and right MC.

#### fNRIS measures as a marker of cortical activation indexed by Δ-HbO*

Cortical deactivation based on Δ-HbO* at either the left or right PFC with their respective cutoff points reached at least 85% sensitivity and 80% specificity in the AUC analysis to discriminate fibromyalgia from controls. Data are shown in Table 3.

**Table 3:** ROC analysis to screening fibromyalgia cortical activation than controls according to the right and left PFC activation time based on the on ΔHbO* value (n = 42).

AUC 95% CI	CI 95%	Cutoffs	Sensitivity	1 − Specificity
Cortical deactivation based on delta value of absolute HbO concentration (ΔHbO*) [HbO concentration before thermal stimuli onset minus HbO concentration level 15 s after thermal stimuli end]on the left PFC (mM)				
0.63	(0.45–0.80)	− 2.0800	1.000	1.000
		− 0.7650	0.950	1.000
		− 0.4250	0.900	1.000
		− 0.3950	0.900	0.938
		− 0.3750	0.900	0.875
		− 0.2800	0.850	0.875
		− 0.1950	0.800	0.875
		− 0.1750	0.800	0.813
Cortical deactivation based on delta value of absolute HbO concentration (ΔHbO*) [HbO concentration before thermal stimuli onset minus HbO concentration level 15 s after thermal stimuli end]on the right PFC (mM)				
0.60	(0.42–0.79)	− 1.6000	1.000	1.000
		− 0.5600	0.950	1.000
		− 0.4750	0.950	0.938
		− 0.3350	0.900	0.938
		− 0.2350	0.900	0.875
		− 0.2050	0.850	0.813

*AUC* area under the curve, *CI* confidence interval, *ROC* receiver operator characteristics.

### Analysis of the relationship between fibromyalgia symptoms and cortical activation after thermal stimuli

Cortical deactivation based on Δ-HbO* values at PFC and MC and their correlations with fibromyalgia symptoms are presented in Table 4. Scatter plots of correlations between the Δ-HbO* values at PFC with scores in the CSS and disability due to pain are presented in Figure 4 of supplementary material.

**Table 4:** Spearman correlation coefficients of the relationship between fibromyalgia symptoms with the delta value of oxyhemoglobin (ΔHbO*) as cortical deactivation measure in either PFC or MC (n = 22).

	(1)	(2)	(3)	(4)	(5)
Portuguese Profile of Chronic Pain Screen (BP-PCP:S) total score (1)	1				
Central Sensitization Inventory (BP-SCI) total score (2)	0.64**	1			
ΔHbO* based on [HbO level before thermal stimuli onset minus HbO level 15 s after thermal stimuli end] on the left PCF (3)	0.40*	0.60**	1		
ΔHbO* based on [HbO level before thermal stimuli onset minus HbO level 15 s after thermal stimuli end] on the right PCF (4)	0.09	0.24	0.80**	1	
ΔHbO* based on [HbO level before thermal stimuli onset minus HbO level 15 s after thermal stimuli end] on the left MC (5)	− 0.06	0.18	0.48*	0.69**	1
ΔHbO* based on [HbO level before thermal stimuli onset minus HbO level 15 s after thermal stimuli end] on the right MC (6)	0.01	0.37	39	0.42	0.73**

Prefrontal cortex (PFC); motor cortex (MC). Correlation is significant at the 0.01 level (2-tailed); ΔHbO* in millimolar (mM).

**Correlation is significant at the 0.05 level (2-tailed).

### Multivariate analysis of the relationship between the severity of fibromyalgia symptoms and PFC assessed by Δ-HbO*

Cortical deactivation based on Δ-HbO* value showed a statistically significant correlation with scores of disabilities due to pain and central sensitization (Table 4). Therefore, we examined their relationship by Generalized Linear Models (GLM). Dependent variables were disability due to pain and central sensitization scores, and the independent variable was Δ-HbO* as a measure of cortical activation at both sides of the PFC. The results of these adjusted multivariate models are presented in Table 5. At the left PFC, a lower Δ-HbO* concentration was positively correlated with disability due to pain and central sensitization symptoms. In contrast, at the right PFC, a lower Δ-HbO* concentration was conversely associated with disability due to pain and central sensitization symptoms.

**Table 5:** Generalized Linear Models of the association between the disability due to pain and central sensitization scores with the ΔHbO peak latency evoked by thermal stimuli on both PFC (n = 22).

	B	Std. error	CI 95%	Wald χ^2^	Df	*P*
**Portuguese Profile of Chronic Pain Screen (BP-PCP:S) total score**						
(Intercept)	68.693	2.908	(62.99 to 74.39)	557.871	1	0.000
Δ-HbO* based on the HbO level before thermal stimuli onset and 15 s after on the left PFC	48.86	15.01	(19.44 to 78.29)	10.598	1	0.001
Δ-HbO* based on the HbO level before thermal stimuli onset and 15 s after on the right PFC	− 21.84	10.56	(− 42.56 to − 1.14)	4.276	1	0.039
**Central Sensitization Inventory (BP-SCI) total score**						
(Intercept)	71.53	1.830	(67.95 to 75.13)	1526.683	1	0.000
Δ-HbO* based on the HbO level before thermal stimuli onset and 15 s after on the left PFC	31.99	9.197	(13.96 to 50.02)	12.09	1	0.00
Δ-HbO* based on the HbO level before thermal stimuli onset and 15 s after on the right PFC	− 15.04	6.441	(− 27.67 to − 2.41)	5.451	1	0.02

Prefrontal cortex (PFC); ΔHbO* in millimolar (mM).

#### Δ-HbO* based on HbO before thermal stimuli onset and 15 s after thermal stimuli onset at PFC distinguishes patients with more disability due to pain and more severe CSS

We conducted a ROC analysis, stratifying for the cutoff points on Δ-HbO* to differentiate fibromyalgia subjects from controls, − 0.175 at the left PFC and − 0.20 at the right PFC (Table 3). The sensitivity, specificity, and AUC using these cutoff points to screen subjects with higher central sensitization symptoms and higher disability due to pain are presented in Table 6.

**Table 6:** ROC analysis to screening the severity of fibromyalgia symptoms according to the right and left PFC activation based on Δ-HbO* (n = 22).

AUC 95% CI	CI 95%	Cutoffs	Sensitivity	1 − Specificity
**Portuguese Profile of Chronic Pain Screen (BP-PCP:S) total score**				
Δ-HbO* based on the HbO level before thermal stimuli onset and 15 s after on the left PFC (mM)				
0.58	(CI 95%, 0.21–0.95)	− 0.175	100	100
Δ-HbO* based on the HbO level before thermal stimuli onset and 15 s after on the right PFC (mM)				
0.59	(CI 95%, 0.20–0.97)	− 0.20	100	100
**Central Sensitization Inventory (BP-SCI) total score**				
Δ-HbO* based on the HbO level before thermal stimuli onset and 15 s after on the left PFC (mM)				
0.82	(CI 95%, 0.61–100)	− 0.175	100	100
Δ-HbO based on the HbO level before thermal stimuli onset and 15 s after on the right PFC (mM)				
0.68	(CI 95%, 0.29–100)	− 0.20	100	100

*AUC* area under the curve, *CI* confidence interval, *ROC* receiver operator characteristics, *mM* millimolar.

## Discussion

This study's main findings highlight that the temperature effect produced a more considerable difference in the absolute concentration of HbO measured by Δ-HbO* at the left PFC in controls compared to fibromyalgia. In contrast, this difference at the left MC was more significant in fibromyalgia than in controls. We found this difference in Δ-HbO* pattern at the left PFC had satisfactory discriminatory properties to differentiate cortical activation in fibromyalgia patients versus controls and discriminate fibromyalgia subjects with more severe CSS symptoms and disability to pain. Also, the peak latency difference within-group revealed that the cortical activation occurred slower at the left MC in fibromyalgia than in controls. In sum, results from the present study suggest that the dynamic measure of HbO changes indexed on peak latency of HbO and Δ-HbO* are sensitive inferential markers to identify cortical dysfunction related to fibromyalgia.

These findings contrast with our initial hypothesis that peak latency and differences in HbO concentration before and after thermal stimuli would be shorter and larger, respectively, in fibromyalgia subjects than in controls exposing that fibromyalgia group would have faster and stronger cortical response. The initial hypothesis was based on the rationale that sustained chronic pain could increase the excitability of pain pathways. The corresponding functional finding would indicate hyperactivation in target areas either as involved in pain processing or used as therapeutic targets to improve pain measures, nominally PFC and MC. Although we found that both cortical activation measures indicated hypoactivation in the left PFC and the left MC in fibromyalgia, the results from the current study are consistent and add information to suggesting that inferential measures based on BOLD signal might be valuable tools to differentiate dysfunctional cortical processing, mainly in areas associated with cognitive and emotional aspects of fibromyalgia, nominally left PFC.

The variability of the BOLD signal that we found in our present study is indirectly coupled with neuro-vascular changes attributed to the intrinsic variability of neural processing, such as synaptic transmission or neurotransmitter functions indirectly assessed by HbO concentration^36^. Such results are not to replace existing diagnostic tools but rather to establish a meaningful neurobiological basis for cortical pain processing to open a new avenue to study the relationship between dysfunction in the neural networks in these areas. In this case, PFC and MC have been used as therapeutic targets for the application of transcranial stimulation, such as tDCS and repetitive TMS. Thus, this model is potentially valuable for differentiating patients from controls or predicting severity of symptoms. However, it is unclear whether it is a model of the relevant clinical pathology at all. Hence, it needs further prospective testing on independent data, since our primary goal is not to provide a complete model of symptoms and behavior but to test hypotheses about structure–function associations based on a well-defined experimental paradigm. Even though the current findings need further validation in longitudinal studies and larger samples, they extend the evidence that fibromyalgia features deteriorated function according to HbO changes across contrasting thermal stimuli. Following this perspective, the larger peak latency difference found in the left MC in fibromyalgia subjects compared with controls might be related to the reduced tone of cortical motor areas. Even though our experimental condition does not assess motor performance, our results agree with the literature showing reduced information processing speed in MC areas in fibromyalgia patients^37,38^. Thus, this result might indicate dysfunctional cortical processes related to fear of movement found in patients with chronic pain^39^ or cognitive problems with impaired motor processing^37,38^. An alternative explanation could be based on a partial inhibition of cortical motor areas during concurrent nociceptive stimulation, which generates a loss of the metabolic advantage in consonance with the severity of symptoms.

Our finding related to maximal activation and average changes in HbO in fibromyalgia subjects compared to controls agrees with a previous study that found lower maximal and mean changes in HbO concentration at both the left and the right PFC in fibromyalgia patients compared to healthy controls during a breath-holding task^40^. Also, another study using fNIRS found reduced brain activity over the frontal regions during a verbal fluency test (VFT) in fibromyalgia patients compared to controls^41^. In earlier studies, a more significant short intracortical inhibition was found in fibromyalgia patients than healthy subjects^4^. Thus, this set of findings suggests reduced PFC activation in fibromyalgia may indicate deterioration in cortical processing function. Although the size of effect related to the group and temperature on cortical activation is only moderate^42^, they support the experimental paradigm used to activate one or more brain target regions involved in pain processing, nominally PFC and MC. These results based on cortical differences of HbO offer a model with consistent predictive properties to discriminate fibromyalgia subjects from controls and to identify fibromyalgia subjects with more severe symptoms, however, caution is warranted before generalizing these findings, since the sample size is small and only one experimental paradigm was tested.

Aligned with this perspective to comprehend the physiology of cortical dynamic processing, the relevance of these findings is to show a framework for understanding the relationship between the function of cortical areas in pain processing and clinical symptoms related to CSS and disability due to pain. In this regard, they open an avenue to identify measures with the potential to be biomarkers, since they integrate the function of cortical areas involved in underlying fibromyalgia symptoms. According to experimental studies, these cortical areas (i.e., PFC and MC ) have been targets to improve pain by non-invasive brain stimulation techniques such as repetitive TMS^43^ and tDCS^44–47^. This effect was showed with extended home-based use of tDCS on the left dorsolateral PFC (DLPFC), which improved pain, psychological symptoms, sleep quality, and disability due to fibromyalgia^44^. Also, previous studies demonstrated that the use of tDCS on the left DLPFC improved attention, working memory, and pain in fibromyalgia^48^ and depressive symptoms in major depressive disorders^49,50^.

In the same way, another study with healthy males using an electrical standardized stimulus on the unilateral right accessory spinal nerve unilateral showed in an integrative framework measures that the fNIRS measures are suitable to comprehend the connections of PFC with the MC in pain processing^51^. Despite limitations in comparing results in these studies because of differences in sex and type of stimulus used in the experimental paradigm, these findings corroborate the role of functional spectroscopic mapping of pain-processing cortical areas related to sensory-discriminative and affective-motivational pain dimensions. Likewise, results of a meta-analysis of studies employing experimental pain stimuli indicate a positive association of the following brain areas with pain processing: primary and secondary somatosensory cortices, insular cortex, ACC, PFC, and thalamus^48^. Thus, these results find support from the anatomic perspective, since the discrimination of pain intensity by the ventral pathway activates the PFC bilaterally, and the spatial discrimination by the dorsal direct path from the posterior parietal cortex triggers the DLPFC activation^40^.

A relevant contribution of our findings is to add results to literature integrating neurophysiological measures with clinical data with the perspective of accelerating the translation of surrogate measures to results to apply at the bedside. The differences in the concentration of Δ-HbO* at the left PFC was positively correlated with disability due to pain and CSS. In contrast, the change in Δ-HbO* at the right PFC by the same thermal stimulus was conversely correlated with the severity of these clinical symptoms (see Table 5). This suggests an imbalance of inter-hemispheric activation, and based on this experimental paradigm, the hypoactivation tends to be more pronounced in the left, whether at MC or PFC. One hypothesis to explain these findings is that some target areas are activated to the detriment of other circuits’ deactivation. This imbalance may be related to functional lateralization of the amygdala in the context of pain. In general, the amygdala’s right central nucleus tends to have a pronociceptive role, while the left has an antinociceptive role^52^. One hypothesis is that the disturbed balance of this system results in chronic pain conditions. Although the explanation for this imbalance is not clear, it may be due to the maintenance of neuronal hyperexcitability in the central left nucleus of the amygdala to counteract the pain-driving effect of the right central nucleus of the amygdala^52^. However, studies related to amygdala lateralization in the context of pain are only beginning, and there are many different elements to consider that will vary significantly based on pain mechanism. We acknowledge that this hypothesis remains relatively broad, but a better comprehension of lateralization of pain processing is relevant to the therapeutic perspective mentioned above. However, the specific intricacies necessary to fully understand how lateralization functions in pain will need further studies that address the side as a variable.

The ROC analysis was used to screen the severity of fibromyalgia symptoms observed after activation of the left PFC based on Δ-HbO*. The cutoff was defined by setting the AUC to offer 100% sensitivity and 98% specificity to screen fibromyalgia patients versus controls (Table 2). It offered an AUC of 0.82 to screen for the severity of CSS. As mentioned above, this result can be a consequence of lateralization of the antinociceptive response. This more considerable change of cortical response in fibromyalgia suggests a deteriorated function of the PFC in pain processing. More precisely, from a conceptual perspective, our findings might explain the pathophysiological processes that underlie fibromyalgia since they integrate the severity of symptoms with dysfunctional changes in cortical areas involved in the cardinal sign of PFC dysfunction, such as cognitive impairment^53^. Additionally, PFC dysfunction might explain the central sensitization-related clinical variables, such as depressive symptoms, insomnia symptoms, perceived level of disability^54^, duration of pain, current pain intensity^55^, average pain intensity, and pain catastrophizing^56^.

We assumed cortical activation changes could not be interpreted as a direct response to nociceptive stimuli since the hemodynamic response is an alternative marker of neuronal activity. Even though we cannot interpret them as a cause–effect response, their contextualized interpretation can help elucidate cortical brain function in targeting areas involved in pain processing and regions targeted by therapeutic approaches. Our findings agree with some of the literature supporting fNIRS measures as a reliably sensitive central measure to comprehend the cortical effect of painful stimulation^36^. Yücel et al. (2015) found that in healthy subjects, the BOLD signals assessed by fNIRS detected a greater activation in M1 upon painful stimulus but no critical changes in PFC. Although many studies have investigated cortical activation using fNIRS in fibromyalgia, a novelty of the current study is the distinct paradigm used^22,57^. Although these results indicate changes by indirect means (metabolic and vascular), they give us new insights to study in real time the effect of different paradigms to evaluate cortical dysfunction related to chronic pain. Additionally, they may be an entry port to assess the complex pain-related neural network and to understand the role of PFC and MC in processing pain signals.

Although these results offer a perspective to comprehend the role of target areas related to pain and emotion using fNIRS that permits an assessment in real time, we need parsimony in their interpretation owing to some limitations in the methods and study design. First, it is a challenge to maintain fibromyalgia patients in the same position for any length of time, and they may even experience scalp pain during hair manipulation. For these reasons, we removed some channels in some cases, and sometimes the quality of the signal was poor. However, we know these difficulties are intrinsic to this type of measure. Second, we identified differences between groups related to age, years of formal education, psychiatric disorders, and medication, several of which are expected. Even though some confounding effects cannot be fully controlled, we found results that indicate differences between groups with biological plausibility. Third, this is a physiological-basis study involving cortical pain processing and given the known difference between sexes in pain processing, we included only female subjects. We understand that this restricts external validity. However, it permitted us to reduce the potential confounding effect of sex on our measures. This is plausible since women are more susceptible to negative emotional responses such as fear of pain^58^, stress, and anxiety^59^. Fourth, we did not find an interaction between group and temperature. Although the explanation is not clear, it might be explained as an error type II. And it is not accessible to compared results among studies that used distinct paradigms to evoke the cortical activation because the kind of stimulus and its intensity is vital to the recruitment of cortical areas. It is important to realize that the cold pressure test is an acute stressor used to measure pain threshold (i.e., first feeling pain). Finally, further research is needed to assess cortical brain activation in chronic pain under different experimental paradigms, such as behavioral tests to determine a cognitive and emotional response, motor tasks, and changes related to effects of different therapeutic approaches (e.g., tDCS, TMS, etc.).

In conclusion, these results indicate that cortical deactivation based on Δ-HbO* at PFC might be a sensitive marker to discriminate fibromyalgia cortical processing and to screen patients with more disability due to pain and more severe CSS. Overall, they offer insight into cortical function in the pathophysiology of primary chronic pain and the modifications of the cortical part of these target areas in response to an effective treatment.

## Supplementary Information

Supplementary Information 1.

Supplementary Information 2.
