# Publisher Correction: The mapping of cortical activation by near-infrared spectroscopy might be a biomarker related to the severity of fibromyalgia symptoms

**DOI:** 10.1038/s41598-021-99824-6

**Published:** 2021-10-22

**Authors:** Daniela Gabiatti Donadel, Maxciel Zortea, Iraci L. S. Torres, Felipe Fregni, Wolnei Caumo

**Affiliations:** 1 Post‑Graduation Program in Medical Sciences, School of Medicine, Universidade Federal do Rio Grande do Sul (UFRGS), Porto Alegre, Brazil; 2 Laboratory of Pain and Neuromodulation at Hospital de Clínicas de Porto Alegre (HCPA), UFRGS, Ramiro Barcelos, 2350, Bairro Rio Branco, Porto Alegre, RS CEP 90035‑003 Brazil; 3 Pharmacology of Pain and Neuromodulation: Pre-Clinical Investigations Research Group at Hospital de Clínicas de Porto Alegre (HCPA), Porto Alegre, Brazil; 4 Department of Neurology, Berenson‑Allen Center for Noninvasive Brain Stimulation, Beth Israel Deaconess Medical Center, Physical Medicine and Rehabilitation, Harvard Medical School, Boston, USA; 5 Pain and Palliative Care Service, Hospital de Clínicas de Porto Alegre (HCPA), Porto Alegre, Brazil; 6 Pain and Anesthesia in Surgery Department, School of Medicine, Universidade Federal do Rio Grande do Sul (UFRGS), Porto Alegre, Brazil

Correction to: *Scientific Reports* 10.1038/s41598-021-94456-2, published online 03 August 2021

The original version of this Article contained errors.

The legends of Figure 1 and 2 were inadvertently switched.

The legend of Figure 1:

“Timeline of the experimental paradigm. Timeline of each assessment pre- and post-thermal stimuli with primary stimulus (at 25 °C) and secondary stimulus (at 5 °C). The cold test was induced by the immersion of the right hand in the water for 30 s or until feeling the first pain sensation with both stimuli and a break of 2 min between each trial.”

now reads:

“The fNIRS' optodes montage. Black dots represent detectors, and white dots represent sources. Left prefrontal cortex—channel 1–9 plus 11 and 12. Right prefrontal cortex—channel 12–20 plus Channel 9 and 10. Right motor cortex—channel 21–30. Left motor cortex—Chanel 31–40.”

The legend of Figure 2:

“The fNIRS' optodes montage. Black dots represent detectors, and white dots represent sources. Left prefrontal cortex—channel 1–9 plus 11 and 12. Right prefrontal cortex—channel 12–20 plus Channel 9 and 10. Right motor cortex—channel 21–30. Left motor cortex—Chanel 31–40.”

now reads:

“Timeline of the experimental paradigm. Timeline of each assessment pre- and post-thermal stimuli with primary stimulus (at 25 °C) and secondary stimulus (at 5 °C). The cold test was induced by the immersion of the right hand in the water for 30 s or until feeling the first pain sensation with both stimuli and a break of 2 min between each trial.”

The Acknowledgements section was omitted and now reads:

We thank the Fundo de Incentivo à Pesquisa e Eventos (FIPE) from the Postgraduate Research Group at the Hospital de Clínicas de Porto Alegre for all the financial support and consultancy services the institution provided since the beginning of the study.

Lastly, the Funding section was incomplete and now reads:

Research funding for Brazilian agencies: (i) Committee for the Development of Higher Education Personnel— CAPES (MZ, post-doctorate scholarship). (ii) National Council for Scientifc and Technological Development— CNPq (Grant Nos. 302688/2017-0 and 420826/2018-1 to W. C.). (iii) Fundo de Incentivo à Pesquisa e Eventos (FIPE) from the Postgraduate Research Group at the Hospital de Clínicas de Porto Alegre (Project No. 2017-0049). Brazilian Innovation Agency (FINEP [Financiadora de Estudos e Projetos]) (process number 1245/13. Fundação de Amparo à Pesquisa do Estado do Rio Grande do Sul (FAPERGS)—Grant No WC, 17/2551-0001 087-6 and 17/2551-0001 476-6). W. C. agrees to be accountable for all aspects of the work, ensuring that questions related to the accuracy or integrity of any part of the work are appropriately investigated and resolved.

The original Article has been corrected.

