# Supplementary material for: The mapping of cortical activation by near-infrared spectroscopy might be a biomarker related to the severity of fibromyalgia symptoms
Source: Sci Rep. 2021 Aug 3;11:15754. doi: 10.1038/s41598-021-94456-2 (PMC8333354; doi:10.1038/s41598-021-94456-2)
Supplement: Supplementary file 1 — Supplementary Information 1. [file 41598_2021_94456_MOESM1_ESM.docx]

**Appendix I**

**The mapping of cortical activation by near-infrared spectroscopy might be a biomarker related to the severity of fibromyalgia symptoms**

Daniela Gabiatti Donadel ^1,2^, Maxciel Zortea^1,2^, Iraci Lucena da Silva Torres^1,3^, Felipe Fregni,^4^ Wolnei Caumo^1,2,5,6^

**Supplementary Table 6.** Relationship of fNIRS channels 1-20 and EEG 10-10 System to compose the channels used.

|  | From | 10-10 System | | To | Side |  | From | 10-10 system | | To | Side |
| --- | --- | --- | --- | --- | --- | --- | --- | --- | --- | --- | --- |
| C1 | Middle Frontal G | F3 | F5 | Middle Frontal G | Left | **C21** | Precentral G | FC6 | C6 | Postcentral G | Right |
| C2 | Middle Frontal G | F3 | F1 | Superior Frontal G | Left | **C22** | Precentral G | FC6 | FC4 | Middle Frontal G | Right |
| C3 | Middle Frontal G | AF3 | F5 | Middle Frontal G | Left | **C23** | Postcentral G | C4 | C6 | Postcentral G | Right |
| C4 | Middle Frontal G | AF7 | Fp1 | Superior Frontal G | Left | **C24** | Postcentral G | C4 | FC4 | Middle Frontal G | Right |
| C5 | Superior Frontal G | AF3 | F1 | Superior Frontal G | Left | **C25** | Postcentral G | C4 | CP4 | Inferior Parietal G | Right |
| C6 | Superior Frontal G | AF3 | Fp1 | Superior Frontal G | Left | **C26** | Postcentral G | C4 | C2 | Precentral G |  |
| C7 | Superior Frontal G | AF3 | AFz | Bilateral Medial | Left | **C27** | Superior Frontal G | FC2 | FC4 | Middle Frontal G | Right |
| C8 | Bilateral Medial | Fz | F1 | Superior Frontal G | Left | **C28** | Postcentral G | FC2 | C2 | Precentral G |  |
| C9 | Bilateral Medial | Fz | AFz | Bilateral Medial | C | **C29** | Postcentral G | CP2 | C6 | Inferior Parietal G | Right |
| C10 | Bilateral Medial | Fz | F2 | Superior Frontal G | Right | **C30** | Postcentral G | CP2 | C2 | Precentral G |  |
| C11 | Bilateral Medial | Fpz | Fp1 | Superior Frontal G | Left | **C31** | Superior Frontal G | FC1 | C1 | Precentral G |  |
| C12 | Bilateral Medial | Fpz | AFz | Bilateral Medial | C | **C32** | Superior Frontal G | FC1 | FC3 | Middle Frontal G | Left |
| C13 | Bilateral Medial | Fpz | FP2 | Superior Frontal G | Right | **C33** | Postcentral G | CP1 | C1 | Precentral G |  |
| C14 | Superior Frontal G | AF4 | AFz | Bilateral Medial | Right | **C34** | Postcentral G | CP1 | CP3 | Inferior Parietal G | Left |
| C15 | Superior Frontal G | AF4 | F2 | Superior Frontal G | Right | **C35** | Postcentral G | C3 | C1 | Precentral G |  |
| C16 | Superior Frontal G | AF4 | Fp2 | Superior Frontal G | Right | **C36** | Postcentral G | C3 | FC3 | Middle Frontal G | Left |
| C17 | Middle Frontal G | F4 | F2 | Superior Frontal G | Right | **C37** | Postcentral G | C3 | CP3 | Inferior Parietal G | Left |
| C18 | Middle Frontal G | F4 | F6 | Middle Frontal G | Right | **C38** | Postcentral G | C3 | C5 | Postcentral G | Left |
| C19 | Middle Frontal G | AF8 | Fp2 | Superior Frontal G | Right | **C39** | Precentral G | FC5 | FC3 | Middle Frontal G | Left |
| C20 | Middle Frontal G | AF8 | F6 | Middle Frontal G | Right | **C40** | Precentral G | FC5 | C5 | Postcentral G | Left |

**DeltaD_PFCE Delta based on peak at 5 - peak at 25**

**DeltaT_PFCE Delta based on peak at 5 - peak at 25**

| Δ Peak LatencyLeft PFC |
| --- |
| Δ Peak LatencyRight PFC |
| Δ’HbO Left PFC |
| Δ’HbO Right PFC |
| Δ Peak LatencyLeft M1 |
| Δ Peak LatencyRight M1 |
| Δ’HbO Left M1 |
| Δ’HbO Right M1 |
