# Supplementary material for: The mapping of cortical activation by near-infrared spectroscopy might be a biomarker related to the severity of fibromyalgia symptoms
Source: Sci Rep. 2021 Aug 3;11:15754. doi: 10.1038/s41598-021-94456-2 (PMC8333354; doi:10.1038/s41598-021-94456-2)
Supplement: Supplementary file 2 — Supplementary Information 2. [file 41598_2021_94456_MOESM2_ESM.pdf]

The mapping of cortical activation by near-infrared spectroscopy might be a biomarker related to the severity of fibromyalgia symptoms

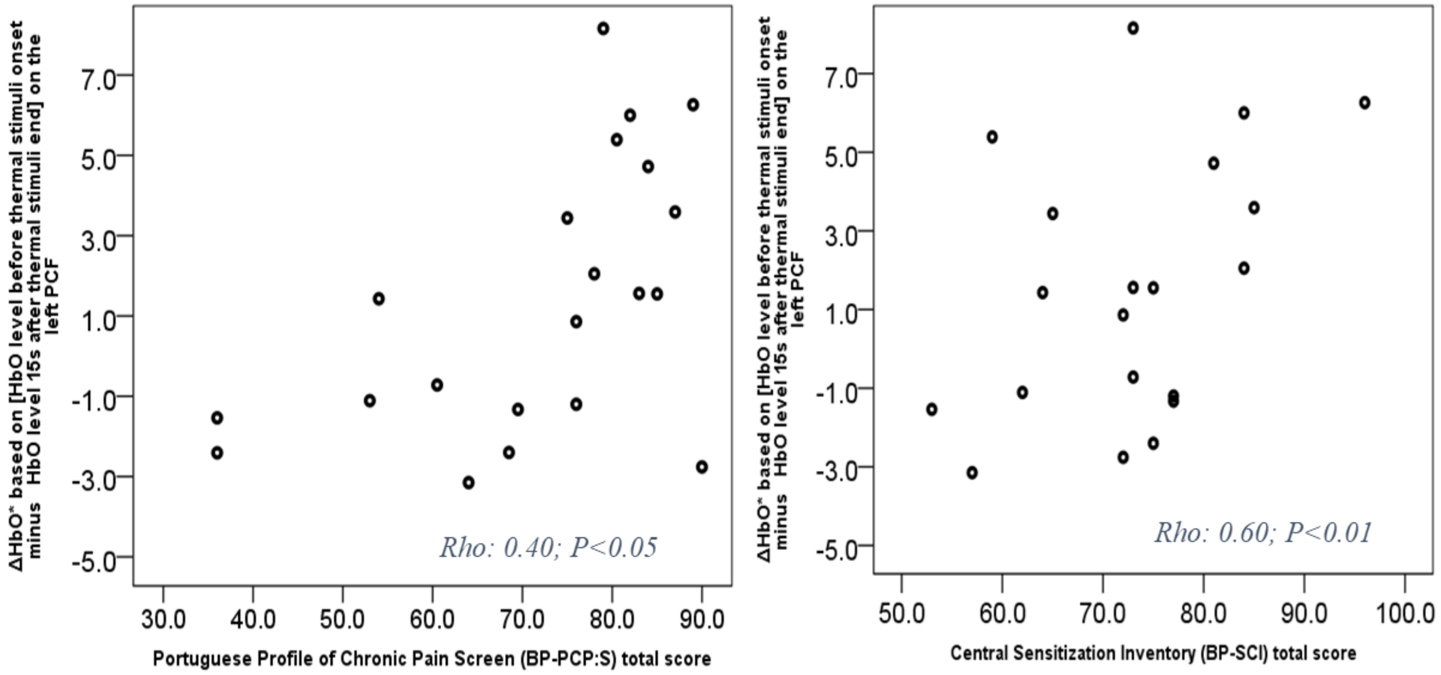

Figure 4. Spearman correlation coefficients of the relationship between the delta value of oxyhemoglobin (ΔHbO*) as cortical activation measure in the left pre-frontal cortex (PFC) and its correlation with the Central Sensitization Score and the score of disability due to pain in the BP-PCP:S (n=22).
